## [Editor Report · eLife Assessment]

This is a **useful** contribution to our understanding of taste perception. The idea that specific receptors function in the pharynx to mediate responses to carboxylic acids is interesting, although the expression analysis is incomplete. Reviewers also have a number of other suggestions for improvement, including the request that authors provide more details about the methodology used. In general, the claims are supported by **solid** evidence and add to a growing body of literature on this topic.

---

## [Referee Report · Reviewer #1 (Public review)]

Summary:

Shrestha et al report an investigation of mechanisms underlying gustatory preference for carboxylic acids in *Drosophila*. They begin with a screen of selected IR mutants, identifying 5 candidates - 2 IR co-receptors and 3 other IRs - whose loss of function causes defects in feeding preference for one or more of the three tested carboxylic acids. The requirement for IR51b, IR94a, and IR94h in carboxylic acid responses is evaluated in more detail using behavior, electrophysiology (labellar sensilla), and calcium imaging (pharyngeal neurons). The behavioral valence of IR94a and IR94h neurons is assessed using optogenetics. Overall the study uses a variety of approaches to test and validate the requirement of IRs in pharyngeal carboxylic acid taste.

Strengths:

The involvement of the identified IRs in gustatory responses to carboxylic acids is very clear from this study. The authors use mutants and transgenic rescue experiments and evaluate outcomes using electrophysiology, behavior, and imaging. Complementary approaches of loss-of-function and artificial activation support the main conclusion that the identified pharyngeal neurons sense carboxylic acids and convey a positive behavioral valence.

Weaknesses:

Some aspects of expression analysis and calcium imaging need to be clarified to better support the conclusions.

(1) The conclusion of two parallel IR-mediated pathways rests on expression analysis of Ir94a-GAL4 and Ir94h-GAL4 lines and the observation that Ir51b expression driven by either can rescue the Ir51b mutant phenotype. However, the expression analysis is not as rigorous as it needs to be for such a conclusion. Prior work found co-expression of Ir94a and Ir94h in the LSO. Here, the co-expression of the two drivers has not been examined, and Ir94a-GAL4 does not appear to be expressed in the LSO. Given the challenges in validating expression patterns in pharyngeal organs, the possibility that the drivers do not entirely capture endogenous expression cannot be ruled out. Rescue experiments using feeding preference or single-cell imaging don't suffice as validation. Plus, the expression of Ir51b could not be defined.

(2) The description of methods and results for the ex vivo calcium imaging is not satisfactory. Details about which cells are being analyzed, and in which organs are not included. No solvent stimulus is tested. The temporal dynamics of the responses are not presented. Movies of the imaging are not included as supplementary information - it would be important to visualize those with what was considered modest movement.

(3) The observed differences in phenotypes of Ir25a and Ir76b mutants are intriguing, as are those between the co-receptor mutants and Ir51b, Ir94a, and Ir94h, but have not been sufficiently considered. Prior studies have also found roles for other response modes (OFF response), other IRs and GRs, and other organs (labellum, tarsi) in behavioral responses to carboxylic acids. Overall, the authors' model may be overly simplistic, and the discussion does not do justice to how their model reconciles with the body of work that already exists.

---

## [Referee Report · Reviewer #2 (Public review)]

Shrestha et al investigated the role of IR receptors in the detection of 3 carboxylic acids in adult *Drosophila*. A low concentration of either of these carboxylic acids added to 2 mM sucrose (1% lactic acid (LA), citric acid (CA), or glycolic acid (GA)) stimulates the consumption of adult flies in choice conditions. The authors use this behavioral test to screen the impact of mutations within 33 receptors belonging to the IR family, a large family of receptors derived from glutamate receptors and expressed both in the olfactory and gustatory sensilla of insects. Within the panel of mutants tested, they observed that 3 receptors (IR25a, IR51b, and IR76b) impaired the detection of LA, CA, and GA, and that 2 others impacted the detection of CA and GA (IR94a and IR94h). Interestingly, impairing IR51b, IR94a, and IR94h did not affect the electrophysiological responses of external gustatory sensilla to LA, CA, and GA. Thanks to the use of GAL4 strains associated with these receptors and thanks to the use of poxn mutants (which do not develop external gustatory sensilla but still have functional internal receptors), they show evidence that IR94a and IR94h are only expressed in two clusters of gustatory neurons of the pharynx, respectively in the VCSO (ventral cibarial sense organ) and in the VCSO + LSO (labral sense organ). As for IR51b, the GAL4 approach was not successful but RT-PCR made on different parts of the insect showed an expression both in the pharyngeal organs and in peripheral receptors. These main findings are then complemented by a host of additional experiments meant to better understand the respective roles of IR94a and IR94h, by using optogenetics and brain calcium imaging using GCamp6. They also report a failed attempt to co-express IR51b, IR94a, and IR94h into external receptors, a co-expression which did not confer the capability of bitter-sensitive cells (expressing GR33a-GAL4) to detect either of the carboxylic acids. These data complete and expand previous observations made on this group and others, and dot to 2 new IR receptors which show an unsuspected specific expression, into organs that still remain difficult to study.

The conclusions of this paper are supported by the data presented, but it remains difficult to make general conclusions as concerns the mechanisms by which carboxylic acids are detected.

(1) All experiments were done with 1% of carboxylic acids. What is the dose dependency of the behavioral responses to these acids, and is it conceivable that other receptors are involved at other concentrations?

(2) One result needs to be better discussed and hypotheses proposed - which is why the mutations of most receptors lead to a loss of detection (mutant flies become incapable of detecting the acid) while mutations in IR94a and IR94h make CA and GA potent deterrents. Does it mean that CA and GA are detected by another set of receptors that, when activated, make flies actively avoid CA and GA? In that case, do the authors think that testing receptors one by one is enough to uncover all the receptors participating in the detection of these substances?

(3) The paper needs to be updated with a recent paper published by Guillemin et al (2024), indicating that LA is detected externally by a combination of IR94e, IR76b and IR25a. IR25a might help to form a fully functional receptor in GR33a neurons (a former study from Chen et al (2017) indicate that IR25a is expressed in all gustatory neurons of the pharynx).

(4) Although it was not the main focus of the paper, it would have been most interesting if the cells expressing IR94a and IR94h were identified, and placed on the functional map proposed by the group of Dahanukar (Chen et al 2017 Cell Reports, Chen et al 2019 Cell Reports).

---

## [Referee Report · Reviewer #3 (Public review)]

Summary:

In this work, the authors investigated the molecular and cellular basis of sour taste perception in *Drosophila melanogaster*, focusing on identifying receptors that mediate attractive responses to certain carboxylic acids. It builds on previous work from the same group that had identified the IR co-receptors IR25a and IR76b for this sensory process, screening a set of mutants in IRs to identify three, IR51b, IR94a, and IR94h, required for feeding preference responses to some or all of the tested acids.

Strengths:

The work is of interest because it assigns sensory roles to IRs of previously unknown function, in particular IR94a and IR94h, and points to pharyngeal neurons in which these receptors are expressed as the relevant sensory neurons (potentially with different roles for IR94a- and IR94h-expressing neurons). The work combines elegant genetics, simple but effective feeding and taste assays, chemo-/opto-genetic activation, and some calcium imaging. Overall the presented data look solid and well-controlled.

Weaknesses:

The in situ expression analysis relies entirely on transgenic driver lines for IR94a and IR94h (which had been previously described, though not fully cited in this work). Importantly, given that many of the behavioral experiments (genetic rescue, physiology, artificial activation) use the IR94a and IR94h GAL4 driver lines, it would be helpful to validate that these faithfully reflect IR94a and IR94h expression (as far as I can tell, such validation wasn't done in the original papers describing these lines as part of a large collection of IR drivers). For IR51b, pharyngeal expression is concluded indirectly from non-quantitative RT-PCR analysis (genetic reporters did not work). The lack of direct detection of gene/protein expression (for example, through RNA FISH, immunofluorescence, or protein tagging) would have made for a more complete characterization of these receptors (for example, there is no direct evidence that they also express IR25a and IR76b, as one might expect). Finally, the relationship of IR94a and IR94h neurons to other types of pharyngeal neurons remains unclear, as are their projection patterns in the SEZ.

Conceptually, the work is of interest mostly to those in the immediate field; there have been a very large number of studies in the past decade (several from this lab) characterizing the contributions of different IRs to various chemosensory processes. The current work doesn't lend much insight into the nature of the minimal functional unit of gustatory IRs (reconstitution of a functional IR in a heterologous neuron/cell has not been achieved here, but this is a limitation of many other previous studies), nor to how different pharyngeal sensory pathways might collaborate to control behavior. Nevertheless, the findings provide a useful contribution to the literature.

---

## [Author Response]

**Reviewer #1 (Public review):**
Summary:Shrestha et al report an investigation of mechanisms underlying gustatory preference for carboxylic acids in *Drosophila*. They begin with a screen of selected IR mutants, identifying 5 candidates - 2 IR co-receptors and 3 other IRs - whose loss of function causes defects in feeding preference for one or more of the three tested carboxylic acids. The requirement for IR51b, IR94a, and IR94h in carboxylic acid responses is evaluated in more detail using behavior, electrophysiology (labellar sensilla), and calcium imaging (pharyngeal neurons). The behavioral valence of IR94a and IR94h neurons is assessed using optogenetics. Overall the study uses a variety of approaches to test and validate the requirement of IRs in pharyngeal carboxylic acid taste.Strengths:The involvement of the identified IRs in gustatory responses to carboxylic acids is very clear from this study. The authors use mutants and transgenic rescue experiments and evaluate outcomes using electrophysiology, behavior, and imaging. Complementary approaches of loss-of-function and artificial activation support the main conclusion that the identified pharyngeal neurons sense carboxylic acids and convey a positive behavioral valence.Weaknesses:Some aspects of expression analysis and calcium imaging need to be clarified to better support the conclusions.(1) The conclusion of two parallel IR-mediated pathways rests on expression analysis of Ir94a-GAL4 and Ir94h-GAL4 lines and the observation that Ir51b expression driven by either can rescue the Ir51b mutant phenotype. However, the expression analysis is not as rigorous as it needs to be for such a conclusion. Prior work found co-expression of Ir94a and Ir94h in the LSO. Here, the co-expression of the two drivers has not been examined, and Ir94a-GAL4 does not appear to be expressed in the LSO. Given the challenges in validating expression patterns in pharyngeal organs, the possibility that the drivers do not entirely capture endogenous expression cannot be ruled out. Rescue experiments using feeding preference or single-cell imaging don't suffice as validation. Plus, the expression of Ir51b could not be defined.

Based on current literature, *Ir94a* and *Ir94h* exhibit distinct expression patterns localized to different sensory regions. Specifically, *Ir94a* is primarily expressed in the V5 region of the VCSO, where it co-localizes with *Ir94c*-*GAL4* (Chen et al., 2017). Conversely, *Ir94h* is found in the L7-7 sensilla of the LSO, where it co-expresses with *Ir94f*, and also within the V2 cells of the VCSO. Notably, the projections of *Ir94a* and *Ir94h* into the dorso-anterior subesophageal ganglion suggest divergent expression patterns rather than co-expression in the pharyngeal regions (Koh et al., 2014). Regarding co-expression of *Ir94a* and *Ir94h* in the LSO, we did not find any evidence to support this claim. Our data reinforce this view, showing that *Ir94a*-*GAL4* expression is limited to the VCSO, while *Ir94h*-*GAL4* is present in both the LSO and VCSO. Thus, the notion of co-expression of *Ir94a* and *Ir94h* in the LSO is not substantiated by current evidence.

As a reviewer suggested, it is possible that the GAL4 drivers utilized may not fully reflect the endogenous expression of these receptors. Despite this limitation, our behavioral, expression, and physiological analyses strongly suggest that *Ir94a* and *Ir94h* are located in distinct regions, supporting a model of two parallel IR-mediated pathways operating within the sensory system.

In addition, RT-PCR analysis confirmed the presence of *Ir51b*. However, due to methodological constraints, we were unable to conduct cell-type-specific expression studies using *Ir51b*-*GAL4*. This limitation, which we have acknowledged in the manuscript, does not detract from our core findings but highlights an area for future research. Further studies utilizing cell-specific expression analysis and co-expression studies with additional drivers could offer more definitive insights into IR51b’s functional role and its interactions within broader IR-mediated pathways.

(2) The description of methods and results for the ex vivo calcium imaging is not satisfactory. Details about which cells are being analyzed, and in which organs are not included. No solvent stimulus is tested. The temporal dynamics of the responses are not presented. Movies of the imaging are not included as supplementary information - it would be important to visualize those with what was considered modest movement.

We appreciate this valuable feedback. As discussed above, *Ir94h* is specifically expressed in the L7-7 sensilla of the LSO, while *Ir94a* is expressed in the V2 cells of the VCSO. This evidence led us to focus specifically on these cells in our calcium imaging study to ensure accuracy and relevance. In our experiments, Adult hemolymph solution (AHL) (108 mM NaCl, 5 mM KCl, 8.2 mM MgCl2, 2 mM CaCl2, 4 mM NaHCO3, 1 mM NaH2PO4, 5 mM HEPES, pH 7.5) was used as the solvent and employed as a pre-stimulus (as mentioned in the Methods section). During this phase, we observed no changes in fluorescence, indicating that AHL itself did not influence the responses. Fluorescence changes occurred only when the test chemical, dissolved in AHL, was introduced. To further confirm that AHL had no impact on the results, we conducted continuous recordings with AHL alone before beginning our main experiments, and these trials confirmed the absence of fluorescence alterations. We have included the temporal dynamics and supplementary video recordings to provide a more comprehensive understanding of our findings.

(3) The observed differences in phenotypes of Ir25a and Ir76b mutants are intriguing, as are those between the co-receptor mutants and Ir51b, Ir94a, and Ir94h, but have not been sufficiently considered. Prior studies have also found roles for other response modes (OFF response), other IRs and GRs, and other organs (labellum, tarsi) in behavioral responses to carboxylic acids. Overall, the authors' model may be overly simplistic, and the discussion does not do justice to how their model reconciles with the body of work that already exists.

Stanley et al. (2021) reported that the gustatory detection of lactic acid requires both IRs and GRs functioning together. Specifically, they found that IR25a mediates the onset peak response (ON response) to lactic acid, while GRs dampen this response and contribute to a removal peak (OFF response). Interestingly, in *Ir25a* mutants, a small onset peak still occurred, while *Gr64a-f* mutants showed an enhanced onset, suggesting that IRs and GRs interact dynamically to modulate taste responses.

In our previous work, we also observed the role of sweet GRs, in addition to *Ir25a* and *Ir76b*, in detecting carboxylic acids in the labellum (Shrestha et al., 2021). This raises the possibility of a similar interplay with carboxylic acids in our current study, where different IRs may contribute to distinct aspects of sensory responses in the pharynx, leading to the phenotypic differences we observed. Moreover, Chen et al. (2017) demonstrated that sour-sensing neurons in the tarsi express both IR76b and IR25a and specifically respond to carboxylic and inorganic acids without reacting to sweet or bitter compounds. This finding points to a specialized role for these receptors in sour detection and suggests a coordinated response involving multiple sensory organs—such as the labellum, tarsi, and pharynx.

The phenotypic differences observed in our mutants align with a more integrated model of carboxylic acid detection, in which multiple receptors and sensory organs contribute to the overall behavioral response. This supports the idea that our current model offers a more detailed understanding of how different carboxylic acids are detected and processed by the gustatory system.

**Reviewer #2 (Public review):**
Shrestha et al investigated the role of IR receptors in the detection of 3 carboxylic acids in adult *Drosophila*. A low concentration of either of these carboxylic acids added to 2 mM sucrose (1% lactic acid (LA), citric acid (CA), or glycolic acid (GA)) stimulates the consumption of adult flies in choice conditions. The authors use this behavioral test to screen the impact of mutations within 33 receptors belonging to the IR family, a large family of receptors derived from glutamate receptors and expressed both in the olfactory and gustatory sensilla of insects. Within the panel of mutants tested, they observed that 3 receptors (IR25a, IR51b, and IR76b) impaired the detection of LA, CA, and GA, and that 2 others impacted the detection of CA and GA (IR94a and IR94h). Interestingly, impairing IR51b, IR94a, and IR94h did not affect the electrophysiological responses of external gustatory sensilla to LA, CA, and GA. Thanks to the use of GAL4 strains associated with these receptors and thanks to the use of poxn mutants (which do not develop external gustatory sensilla but still have functional internal receptors), they show evidence that IR94a and IR94h are only expressed in two clusters of gustatory neurons of the pharynx, respectively in the VCSO (ventral cibarial sense organ) and in the VCSO + LSO (labral sense organ). As for IR51b, the GAL4 approach was not successful but RT-PCR made on different parts of the insect showed an expression both in the pharyngeal organs and in peripheral receptors. These main findings are then complemented by a host of additional experiments meant to better understand the respective roles of IR94a and IR94h, by using optogenetics and brain calcium imaging using GCamp6. They also report a failed attempt to co-express IR51b, IR94a, and IR94h into external receptors, a co-expression which did not confer the capability of bitter-sensitive cells (expressing GR33a-GAL4) to detect either of the carboxylic acids. These data complete and expand previous observations made on this group and others, and dot to 2 new IR receptors which show an unsuspected specific expression, into organs that still remain difficult to study.The conclusions of this paper are supported by the data presented, but it remains difficult to make general conclusions as concerns the mechanisms by which carboxylic acids are detected.(1) All experiments were done with 1% of carboxylic acids. What is the dose dependency of the behavioral responses to these acids, and is it conceivable that other receptors are involved at other concentrations?

In our study, we conducted experiments to examine the dose dependency of behavioral responses to carboxylic acids, with results presented in Supplementary Figure 1. We found that lower concentrations of carboxylic acids are perceived as attractive, while higher concentrations are aversive. This differential response suggests that the receptors identified in our study are primarily tuned to detect low concentrations of these acids. Since higher concentrations elicited aversive responses, it is plausible that additional receptors, beyond the scope of our study, may be involved in sensing these higher concentrations. These receptors could be part of other gustatory receptor neurons that respond specifically to increased acid levels, as fruit flies tend to avoid higher concentrations. We propose that future research could investigate these alternative pathways to gain a complete understanding of the behavioral responses to carboxylic acids. In summary, our findings suggest that specific receptors are involved in detecting low concentrations, while distinct receptor pathways—possibly mediated by other GRNs—may regulate responses to higher concentrations.

(2) One result needs to be better discussed and hypotheses proposed - which is why the mutations of most receptors lead to a loss of detection (mutant flies become incapable of detecting the acid) while mutations in IR94a and IR94h make CA and GA potent deterrents. Does it mean that CA and GA are detected by another set of receptors that, when activated, make flies actively avoid CA and GA? In that case, do the authors think that testing receptors one by one is enough to uncover all the receptors participating in the detection of these substances?

As we mentioned above, it is possible that distinct receptor pathways mediate avoidance of GA and CA. This suggests that CA and GA might activate different sets of receptors that trigger avoidance behavior, pointing to a more complex interplay of receptor activity than we initially considered. Certain acids may indeed be detected by multiple receptors, with each receptor contributing uniquely to the behavioral response. Regarding the sufficiency of testing receptors individually, we recognize the limitations of this approach. Examining receptors one by one may not reveal the full spectrum of receptors involved, especially due to potential interactions or compensatory mechanisms that only emerge when certain receptors are inactive. Therefore, a more holistic approach—such as genetic screens for behavioral responses or using complex genetic models to disrupt multiple receptors simultaneously—could provide deeper insights. Moving forward, incorporating receptor interactions that modulate each other, along with more comprehensive assays, could help explain these discrepancies by uncovering previously overlooked receptor functions.

(3) The paper needs to be updated with a recent paper published by Guillemin et al (2024), indicating that LA is detected externally by a combination of IR94e, IR76b and IR25a. IR25a might help to form a fully functional receptor in GR33a neurons (a former study from Chen et al (2017) indicate that IR25a is expressed in all gustatory neurons of the pharynx).

According to Guillemin et al. (2024), the combination of IR94e, IR76b, and IR25a is required for amino acid detection but not for detecting lactic acid (LA). In their calcium imaging experiments, 100 mM LA elicited a response similar to the vehicle control, suggesting that these receptors do not play a role in LA detection.

(4) Although it was not the main focus of the paper, it would have been most interesting if the cells expressing IR94a and IR94h were identified, and placed on the functional map proposed by the group of Dahanukar (Chen et al 2017 Cell Reports, Chen et al 2019 Cell Reports).

The expression patterns of IR94a and IR94h were previously detailed by Chen et al. (2017), showing that IR94h is expressed in the labial sense organ (LSO, specifically in L7-7) and the ventral cibarial sense organ (VCSO, V2), while IR94a is expressed in the VCSO (V5). Given this established information, we referenced these known expression patterns without replicating the mapping in our study. Our primary focus was to investigate the functional role of these neurons within the pharynx, and we believe we have successfully highlighted their specific contributions. However, we recognize that integrating the functional mapping of these neurons in alignment with the work of Dahanukar’s group would have strengthened our findings and provided a more comprehensive understanding. We acknowledge this as a limitation of our study and appreciate your suggestion, as it points to a valuable direction for future research.

**Reviewer #3 (Public review):**
Summary:In this work, the authors investigated the molecular and cellular basis of sour taste perception in *Drosophila melanogaster*, focusing on identifying receptors that mediate attractive responses to certain carboxylic acids. It builds on previous work from the same group that had identified the IR co-receptors IR25a and IR76b for this sensory process, screening a set of mutants in IRs to identify three, IR51b, IR94a, and IR94h, required for feeding preference responses to some or all of the tested acids.Strengths:The work is of interest because it assigns sensory roles to IRs of previously unknown function, in particular IR94a and IR94h, and points to pharyngeal neurons in which these receptors are expressed as the relevant sensory neurons (potentially with different roles for IR94a- and IR94h-expressing neurons). The work combines elegant genetics, simple but effective feeding and taste assays, chemo-/opto-genetic activation, and some calcium imaging. Overall the presented data look solid and well-controlled.Weaknesses:The in situ expression analysis relies entirely on transgenic driver lines for IR94a and IR94h (which had been previously described, though not fully cited in this work). Importantly, given that many of the behavioral experiments (genetic rescue, physiology, artificial activation) use the IR94a and IR94h GAL4 driver lines, it would be helpful to validate that these faithfully reflect IR94a and IR94h expression (as far as I can tell, such validation wasn't done in the original papers describing these lines as part of a large collection of IR drivers). For IR51b, pharyngeal expression is concluded indirectly from non-quantitative RT-PCR analysis (genetic reporters did not work). The lack of direct detection of gene/protein expression (for example, through RNA FISH, immunofluorescence, or protein tagging) would have made for a more complete characterization of these receptors (for example, there is no direct evidence that they also express IR25a and IR76b, as one might expect). Finally, the relationship of IR94a and IR94h neurons to other types of pharyngeal neurons remains unclear, as are their projection patterns in the SEZ.Conceptually, the work is of interest mostly to those in the immediate field; there have been a very large number of studies in the past decade (several from this lab) characterizing the contributions of different IRs to various chemosensory processes. The current work doesn't lend much insight into the nature of the minimal functional unit of gustatory IRs (reconstitution of a functional IR in a heterologous neuron/cell has not been achieved here, but this is a limitation of many other previous studies), nor to how different pharyngeal sensory pathways might collaborate to control behavior. Nevertheless, the findings provide a useful contribution to the literature.

We appreciate your thoughtful feedback. As noted in our response, our primary objective was to investigate the sensory functions of IR94a and IR94h. To this end, we conducted behavioral assays, which we validated with additional approaches including genetic rescue, physiological tests, and artificial activation. Throughout these experiments, we extensively utilized *Ir94a*- and *Ir94h*-*GAL4* driver lines. To ensure these lines accurately reflect the expression of IR94a and IR94h, we verified their expression patterns using immunohistochemistry across various body parts. Our results align with previous findings that show both receptors are exclusively expressed in the pharynx. Regarding IR51b, we employed RT-PCR due to its high sensitivity and specificity, which supported our hypothesis. Nonetheless, we agree that more direct detection methods would have provided a stronger validation of IR51b expression. Our previous study (Sang et al., 2024) also demonstrated the pharyngeal expression of co-expressed receptors, specifically IR25a and IR76b. However, we recognize that the lack of direct evidence for their co-expression with IR51b remains a significant gap. This limitation primarily stems from the unavailability of specific reagents needed for direct assays targeting IR51b, which restricted our experimental approach.

You also raised the potential relationship between IR94a and IR94h neurons and other pharyngeal neuron types, including their projection patterns in the subesophageal zone. This is indeed an important area for future research that could clarify neural connectivity and further our understanding of sensory mechanisms. However, our study was focused on exploring sensory mechanisms in peripheral regions rather than detailed neural mapping in the SEZ. Investigating these connections would undoubtedly provide valuable insights into the neural circuitry involved and represents an intriguing direction for future research.