# Supplementary figures and images for: Pharyngeal neuronal mechanisms governing sour taste perception in Drosophila melanogaster
Source: eLife. 2024 Dec 11;13:RP101439. doi: 10.7554/eLife.101439 (PMC11634064; doi:10.7554/eLife.101439)

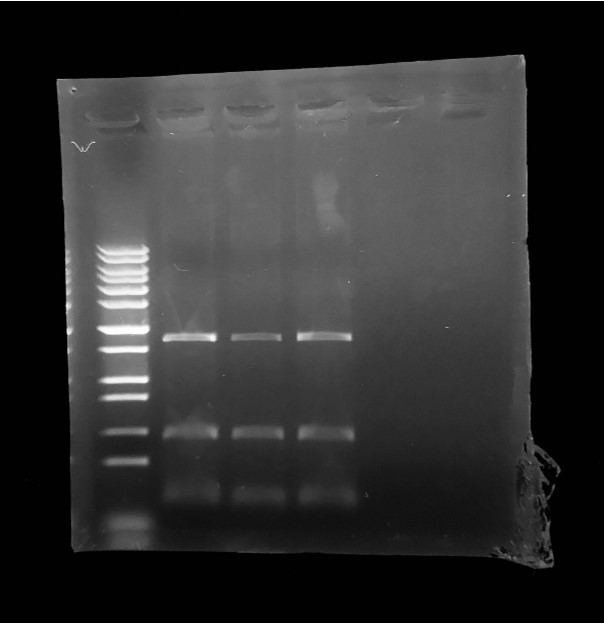

Supplement: Figure 3—source data 1. — Given gel is the picture of RT-PCR resulting Ir51b expression in whole body, labellum, and pharynx. [file elife-101439-fig3-data1.zip › Original files for gel picture displayed in Figure 3E..jpg]

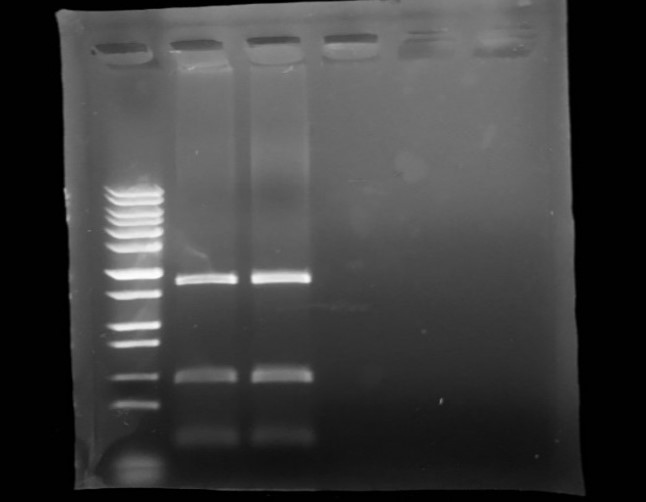

Supplement: Figure 3—source data 1. — Given gel is the picture of RT-PCR resulting Ir51b expression in whole body, labellum, and pharynx. [file elife-101439-fig3-data1.zip › Original files for gel picture displayed in Figure 3F..jpg]
