# Supplementary material for: Pharyngeal neuronal mechanisms governing sour taste perception in Drosophila melanogaster
Source: eLife. 2024 Dec 11;13:RP101439. doi: 10.7554/eLife.101439 (PMC11634064; doi:10.7554/eLife.101439)
Supplement: Figure 3—source data 2. — Given gel is the picture of results from RT-PCR showing Ir51b expression in control (w1118) and poxn null alleles (poxn70 and poxnΔM22−B5) from pharynx. [file elife-101439-fig3-data2.zip › Figure 3_Source data1/PDF file containing gel picture for Figure 3E, indicating the relevant bands..pdf]

Raw photograph file of Figure 3E

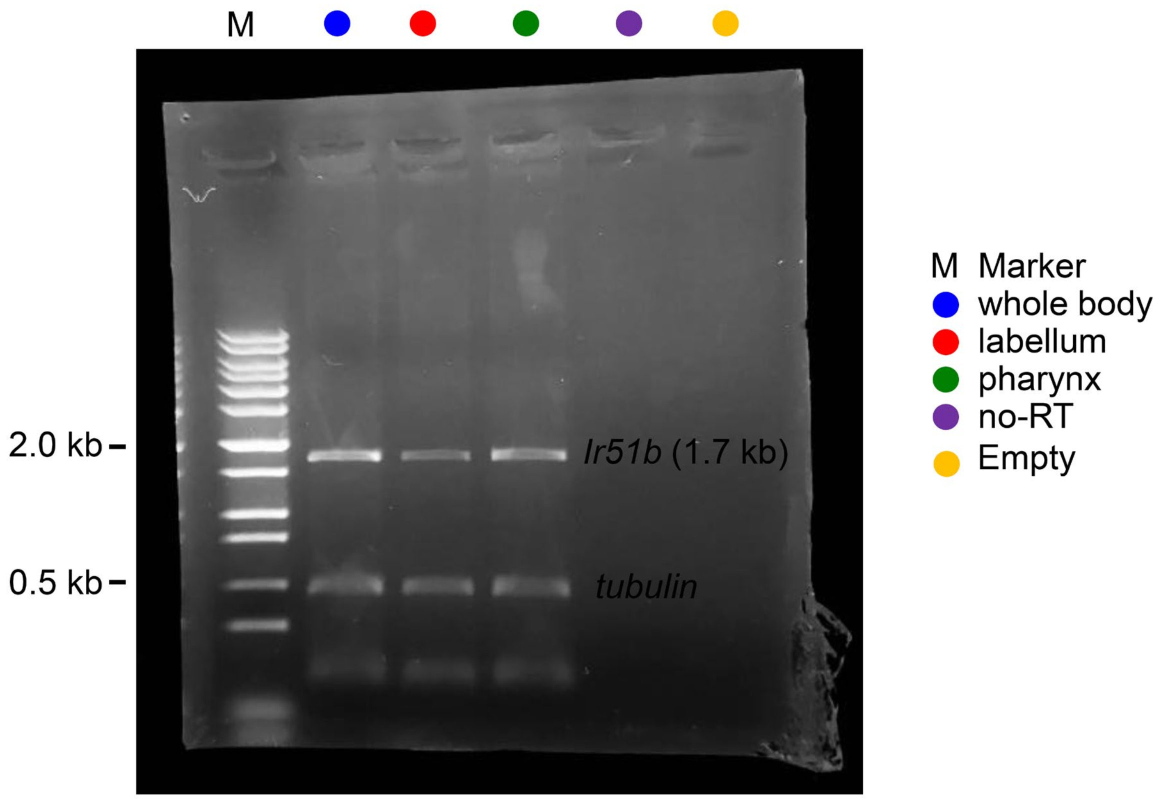

Figure 3—Source Data 2. Original gel picture to Figure 3, panel E. Given gel is the picture of RT-PCR resulting *Ir51b* expression in whole body, labellum, and pharynx. In lanes designated as "no-RT," polyA+ RNA extracts underwent RT omission, and the absence of PCR products serves as evidence of the absence of genomic DNA contamination. Amplified tubulin (0.5 kb) was used as control. DNA ladder marker is denoted by "M."" Dots color represents respective organs indicated in the legend section. (lanes 6; is empty, not shown in figure 3E).
