# Supplementary material for: Pharyngeal neuronal mechanisms governing sour taste perception in Drosophila melanogaster
Source: eLife. 2024 Dec 11;13:RP101439. doi: 10.7554/eLife.101439 (PMC11634064; doi:10.7554/eLife.101439)
Supplement: Supplementary file 1. [file elife-101439-supp1.docx]

***Supplementary File 1*.** Statistics for the data shown in ***Figure 2—figure supplement 1A***

| **Genotype** | | **PI** | | | | |  |
| --- | --- | --- | --- | --- | --- | --- | --- |
|  |  | **0.1%**  **LA** | **0.5%**  **LA** | **1%**  **LA** | **5%**  **LA** | **10%**  **LA** |  |
| control | 0.14 ± 0.05 | | 0.66 ± 0.05 | 0.67 ± 0.08 | 0.56 ± 0.04 | 0.16 ± 0.10 |  |
| *Ir25a^2^* | -0.19 ± 0.06 | | 0.18 ± 0.03 | 0.20 ± 0.03 | 0.14 ± 0.06 | -0.06 ± 0.02 |  |
| *Ir51b^1^* | -0.27 ± 0.05 | | 0.14 ± 0.01 | 0.17 ± 0.04 | 0.13 ± 0.04 | -0.03 ± 0.07 |  |
| *Ir76b^1^* | -0.20 ± 0.07 | | 0.19 ± 0.05 | 0.25 ± 0.07 | 0.14 ± 0.02 | 0.00 ± 0.03 |  |
| *Ir94a^1^* | 0.13 ± 0.04 | | 0.52 ± 0.06 | 0.50 ± 0.04 | 0.45 ± 0.06 | -0.05 ± 0.05 |  |
| *Ir94h^1^* | 0.15 ± 0.06 | | 0.51 ± 0.03 | 0.52 ± 0.02 | 0.43 ± 0.07 | -0.04 ± 0.15 |  |
|  | | ***P* values** | | | | |  |
| control | | - | | - | - | - | - |
| *Ir25a^2^* | | 0.010^¶^ | | 4.40×10^-7^ | 5.06×10^-5^ | 3.65×10^-4^ | 0.603 |
| *Ir51b^1^* | | 0.001 | | 9.32×10^-8^ | 2.02×10^-5^ | 3.18×10^-4^ | 0.737 |
| *Ir76b^1^* | | 0.009 | | 6.99×10^-7^ | 2.83×10^-4^ | 3.56×10^-4^ | 0.861 |
| *Ir94a^1^* | | 1.000* | | 0.315 | 0.451 | 0.846 | 0.651 |
| *Ir94h^1^* | | 1.000* | | 0.234 | 0.536 | 0.700 | 0.709 |

*( * marks represents greater than 0.9999 P value,* ^¶^*0.0097)*

| **Genotype** | | **PI** | | | | |  |
| --- | --- | --- | --- | --- | --- | --- | --- |
|  |  | **0.1%**  **CA** | **0.5%**  **CA** | **1%**  **CA** | **5%**  **CA** | **10%**  **CA** |  |
| control | -0.03 ± 0.03 | | 0.44 ± 0.04 | 0.68 ± 0.07 | 0.08 ± 0.03 | -0.52 ± 0.07 |  |
| *Ir25a^2^* | -0.29 ± 0.08 | | -0.24 ± 0.05 | 0.01 ± 0.03 | -0.49 ± 0.08 | -0.51 ± 0.05 |  |
| *Ir51b^1^* | -0.26 ± 0.02 | | -0.14 ± 0.04 | 0.25 ± 0.09 | -0.30 ± 0.09 | -0.48 ± 0.06 |  |
| *Ir76b^1^* | -0.30 ± 0.07 | | -0.12 ± 0.06 | -0.04 ± 0.06 | -0.21 ± 0.05 | -0.46 ± 0.07 |  |
| *Ir94a^1^* | -0.35 ± 0.08 | | -0.17 ± 0.08 | 0.00± 0.02 | -0.25 ± 0.08 | -0.52 ± 0.05 |  |
| *Ir94h^1^* | -0.30 ± 0.08 | | -0.20 ± 0.05 | -0.47 ± 0.11 | -0.63 ± 0.06 | -0.70 ± 0.07 |  |
|  | | ***P* values** | | | | |  |
| control | | - | | - | - | - | - |
| *Ir25a^2^* | | 0.230 | | 1.80×10^-7^ | 2.27×10^-5^ | 2.44×10^-4^ | 1.000* |
| *Ir51b^1^* | | 0.362 | | 3.89×10^-6^ | 0.009 | 0.023 | 1.000* |
| *Ir76b^1^* | | 0.194 | | 7.06×10^-6^ | 6.21×10^-6^ | 0.050^¶^ | 0.996 |
| *Ir94a^1^* | | 0.084 | | 1.63×10^-6^ | 2.06×10^-6^ | 0.049 | 1.000* |
| *Ir94h^1^* | | 0.217 | | 5.71×10^-7^ | 2.46×10^-10^ | 6.39×10^-6^ | 0.498 |

*( * marks represents greater than 0.9999 P value, ^¶^0.0496)*

| **Genotype** | | **PI** | | | | |  |
| --- | --- | --- | --- | --- | --- | --- | --- |
|  |  | **0.1%**  **GA** | **0.5%**  **GA** | **1%**  **GA** | **5%**  **GA** | **10%**  **GA** |  |
| control | 0.09 ± 0.04 | | 0.54 ± 0.05 | 0.56 ± 0.05 | -0.43 ± 0.03 | -0.59 ± 0.02 |  |
| *Ir25a^2^* | -0.39 ± 0.09 | | -0.11 ± 0.03 | -0.10 ± 0.06 | -0.35 ± 0.09 | -0.40 ± 0.07 |  |
| *Ir51b^1^* | -0.40 ± 0.07 | | 0.14 ± 0.08 | 0.12 ± 0.05 | -0.34 ± 0.10 | -0.66 ± 0.10 |  |
| *Ir76b^1^* | -0.31 ± 0.05 | | -0.28 ± 0.05 | -0.42 ± 0.10 | -0.49 ± 0.09 | -0.50 ± 0.09 |  |
| *Ir94a^1^* | -0.44 ± 0.08 | | -0.34 ± 0.10 | -0.29 ± 0.04 | -0.60 ± 0.08 | -0.64 ± 0.10 |  |
| *Ir94h^1^* | -0.45 ± 0.09 | | -0.31 ± 0.08 | -0.38 ± 0.06 | -0.56 ± 0.08 | -0.58 ± 0.06 |  |
|  | | ***P* values** | | | | |  |
| control | | - | | - | - | - | - |
| *Ir25a^2^* | | 0.005 | | 3.73×10^-5^ | 5.78×10^-6^ | 0.990 | 0.737 |
| *Ir51b^1^* | | 0.003 | | 0.020 | 0.003 | 0.985 | 0.996 |
| *Ir76b^1^* | | 0.024 | | 5.06×10^-7^ | 1.97×10^-9^ | 0.998 | 0.985 |
| *Ir94a^1^* | | 0.001 | | 1.25×10^-7^ | 4.16×10^-8^ | 0.788 | 1.000* |
| *Ir94h^1^* | | 9.58×10^-5^ | | 2.17×10^-7^ | 5.50×10^-9^ | 0.925 | 1.000* |

*( * marks represents greater than 0.9999 P value)*
