# Supplementary material for: Pharyngeal neuronal mechanisms governing sour taste perception in Drosophila melanogaster
Source: eLife. 2024 Dec 11;13:RP101439. doi: 10.7554/eLife.101439 (PMC11634064; doi:10.7554/eLife.101439)
Supplement: Supplementary file 2. [file elife-101439-supp2.docx]

***Supplementary File 2*.** Statistics for the data shown in ***Figure 2—figure supplement 1B***

| **Genotype** | | **PI** | | | | |  |
| --- | --- | --- | --- | --- | --- | --- | --- |
|  |  | **0.1%**  **LA** | **0.5%**  **LA** | **1%**  **LA** | **5%**  **LA** | **10%**  **LA** |  |
| control | 0.10 ± 0.10 | | 0.64 ± 0.04 | 0.66 ± 0.06 | 0.27 ± 0.07 | 0.03 ± 0.05 |  |
| *Ir25a^2^* | -0.14 ± 0.10 | | -0.02 ± 0.07 | -0.02 ± 0.03 | -0.10 ± 0.03 | -0.18 ± 0.03 |  |
| *Ir51b^1^* | -0.24 ± 0.08 | | -0.09 ± 0.03 | -0.04 ± 0.04 | -0.06 ± 0.03 | -0.20 ± 0.10 |  |
| *Ir76b^1^* | 0.09 ± 0.02 | | 0.11 ± 0.06 | 0.09 ± 0.05 | -0.03 ± 0.07 | -0.18 ± 0.04 |  |
| *Ir94a^1^* | 0.07 ± 0.05 | | 0.59 ± 0.05 | 0.53 ± 0.04 | 0.25 ± 0.03 | 0.01 ± 0.05 |  |
| *Ir94h^1^* | 0.06 ± 0.07 | | 0.52 ± 0.08 | 0.53 ± 0.05 | 0.17 ± 0.06 | 0.01 ± 0.07 |  |
|  | | ***P* values** | | | | |  |
| control | | - | | - | - | - | - |
| *Ir25a^2^* | | 0.459 | | 6.08×10^-7^ | 3.70×10^-9^ | 0.002 | 0.318 |
| *Ir51b^1^* | | 0.122 | | 8.61×10^-8^ | 1.90×10^-9^ | 0.005 | 0.228 |
| *Ir76b^1^* | | 1.000* | | 4.37×10^-5^ | 1.67×10^-7^ | 0.009 | 0.092 |
| *Ir94a^1^* | | 1.000* | | 0.995 | 0.585 | 1.000* | 1.000* |
| *Ir94h^1^* | | 1.000* | | 0.831 | 0.544 | 0.878 | 1.000* |

*( * marks represents greater than 0.9999 P value)*

| **Genotype** | | **PI** | | | | |  |
| --- | --- | --- | --- | --- | --- | --- | --- |
|  |  | **0.1%**  **CA** | **0.5%**  **CA** | **1%**  **CA** | **5%**  **CA** | **10%**  **CA** |  |
| control | -0.04 ± 0.04 | | 0.42 ± 0.12 | 0.66 ± 0.13 | 0.38 ± 0.11 | -0.15 ± 0.07 |  |
| *Ir25a^2^* | -0.09 ± 0.07 | | -0.31 ± 0.10 | -0.21 ± 0.03 | -0.30 ± 0.04 | -0.37 ± 0.07 |  |
| *Ir51b^1^* | -0.29 ± 0.06 | | -0.31 ± 0.09 | -0.19 ± 0.09 | -0.24 ± 0.04 | -0.32 ± 0.08 |  |
| *Ir76b^1^* | -0.29 ± 0.05 | | -0.10 ± 0.06 | -0.05 ± 0.06 | -0.06 ± 0.04 | -0.22 ± 0.05 |  |
| *Ir94a^1^* | -0.26 ± 0.14 | | -0.28 ± 0.08 | 0.27 ± 0.11 | -0.11 ± 0.11 | -0.27 ± 0.03 |  |
| *Ir94h^1^* | -0.27 ± 0.06 | | -0.06 ± 0.07 | -0.08 ± 0.03 | -0.11 ± 0.04 | -0.32 ± 0.02 |  |
|  | | ***P* values** | | | | |  |
| control | | - | | - | - | - | - |
| *Ir25a^2^* | | 1.000* | | 2.86×10^-4^ | 9.84×10^-6^ | 3.70×10^-5^ | 0.280 |
| *Ir51b^1^* | | 0.402 | | 2.68×10^-4^ | 1.32×10^-5^ | 1.61×10^-4^ | 0.577 |
| *Ir76b^1^* | | 0.393 | | 0.018 | 2.77×10^-4^ | 0.013 | 0.981 |
| *Ir94a^1^* | | 0.533 | | 5.06×10^-4^ | 2.55×10^-6^ | 0.004 | 0.828 |
| *Ir94h^1^* | | 0.518 | | 0.032 | 1.38×10^-4^ | 0.004 | 0.539 |

*( * marks represents greater than 0.9999 P value)*

| **Genotype** | | **PI** | | | | |  |
| --- | --- | --- | --- | --- | --- | --- | --- |
|  |  | **0.1%**  **GA** | **0.5%**  **GA** | **1%**  **GA** | **5%**  **GA** | **10%**  **GA** |  |
| control | 0.14 ± 0.07 | | 0.59 ± 0.08 | 0.62 ± 0.11 | -0.42 ± 0.05 | -0.49 ± 0.09 |  |
| *Ir25a^2^* | -0.10 ± 0.05 | | -0.00 ± 0.04 | -0.13 ± 0.02 | -0.20 ± 0.07 | -0.32 ± 0.03 |  |
| *Ir51b^1^* | -0.14 ± 0.05 | | 0.13 ± 0.10 | 0.09 ± 0.03 | -0.18 ± 0.03 | -0.30 ± 0.06 |  |
| *Ir76b^1^* | 0.02 ± 0.10 | | 0.13 ± 0.06 | 0.13 ± 0.03 | -0.21 ± 0.06 | -0.37 ± 0.11 |  |
| *Ir94a^1^* | -0.15 ± 0.09 | | -0.15 ± 0.08 | -0.14 ± 0.08 | -0.36 ± 0.04 | -0.51 ± 0.03 |  |
| *Ir94h^1^* | -0.16 ± 0.09 | | 0.13 ± 0.09 | -0.07 ± 0.06 | -0.25 ± 0.03 | -0.43 ± 0.02 |  |
|  | | ***P* values** | | | | |  |
| control | | - | | - | - | - | - |
| *Ir25a^2^* | | 0.469 | | 9.06×10^-4^ | 1.19×10^-6^ | 0.128 | 0.646 |
| *Ir51b^1^* | | 0.281 | | 0.016 | 4.21×10^-4^ | 0.071 | 0.482 |
| *Ir76b^1^* | | 0.944 | | 0.017 | 0.001 | 0.154 | 0.869 |
| *Ir94a^1^* | | 0.267 | | 3.16×10^-5^ | 9.69×10^-7^ | 0.988 | 1.000* |
| *Ir94h^1^* | | 0.231 | | 0.015 | 5.51×10^-6^ | 0.347 | 0.994 |

*( * marks represents greater than 0.9999 P value)*
