# Supplementary material for: Pharyngeal neuronal mechanisms governing sour taste perception in Drosophila melanogaster
Source: eLife. 2024 Dec 11;13:RP101439. doi: 10.7554/eLife.101439 (PMC11634064; doi:10.7554/eLife.101439)
Supplement: Supplementary file 3. [file elife-101439-supp3.docx]

***Supplementary File 3*.** Statistics for the data shown in ***Figure 2—figure supplement 1C***

| **Genotype** | | **PI** | | | | |
| --- | --- | --- | --- | --- | --- | --- |
|  |  | **2.69**  **LA** | **3.50**  **LA** | **5.00**  **LA** | **7.00**  **LA** |  |
| control | 0.66 ± 0.04 | | 0.56 ± 0.03 | 0.22 ± 0.06 | 0.12 ± 0.09 |  |
| *Ir25a^2^* | 0.16 ± 0.03 | | 0.13 ± 0.05 | 0.09 ± 0.06 | -0.09 ± 0.05 |  |
| *Ir51b^1^* | 0.04 ± 0.03 | | 0.00 ± 0.05 | -0.01 ± 0.05 | -0.04 ± 0.06 |  |
| *Ir76b^1^* | 0.10 ± 0.02 | | 0.04 ± 0.08 | 0.03 ± 0.07 | -0.07 ± 0.07 |  |
| *Ir94a^1^* | 0.55 ± 0.03 | | 0.51 ± 0.02 | 0.20 ± 0.03 | 0.15 ± 0.05 |  |
| *Ir94h^1^* | 0.57 ± 0.03 | | 0.51 ± 0.01 | 0.22 ± 0.03 | 0.15 ± 0.05 |  |
|  | | ***P* values** | | | | |
| control | | - | | - | - | - |
| *Ir25a^2^* | | 7.82×10^-11^ | | 1.40×10^-5^ | 0.728 | 0.412 |
| *Ir51b^1^* | | 3.51×10^-13^ | | 7.83×10^-8^ | 0.105 | 0.721 |
| *Ir76b^1^* | | 5.18×10^-12^ | | 2.80×10^-7^ | 0.263 | 0.541 |
| *Ir94a^1^* | | 0.268 | | 0.991 | 1.000* | 1.000*** |
| *Ir94h^1^* | | 0.493 | | 0.987 | 1.000* | 1.000*** |

*( * marks represents greater than 0.9999 P value)*

| **Genotype** | | **PI** | | | | |
| --- | --- | --- | --- | --- | --- | --- |
|  |  | **2.27**  **CA** | **3.50**  **CA** | **5.00**  **CA** | **7.00**  **CA** |  |
| control | 0.68 ± 0.07 | | 0.66 ± 0.07 | 0.13 ± 0.04 | -0.43 ± 0.10 |  |
| *Ir25a^2^* | 0.00 ± 0.06 | | 0.10 ± 0.07 | -0.09 ± 0.06 | -0.29 ± 0.08 |  |
| *Ir51b^1^* | 0.02 ± 0.04 | | -0.07 ± 0.10 | -0.13 ± 0.08 | -0.22 ± 0.03 |  |
| *Ir76b^1^* | -0.25 ± 0.03 | | 0.20 ± 0.05 | -0.01 ± 0.04 | -0.25 ± 0.07 |  |
| *Ir94a^1^* | 0.00 ± 0.05 | | -0.01 ± 0.03 | -0.12 ± 0.06 | -0.27 ± 0.04 |  |
| *Ir94h^1^* | -0.55 ± 0.14 | | -0.08 ± 0.05 | -0.11 ± 0.09 | -0.26 ± 0.07 |  |
|  | | ***P* values** | | | | |
| control | | - | | - | - | - |
| *Ir25a^2^* | | 7.86×10^-5^ | | 1.53×10^-4^ | 0.367 | 0.829 |
| *Ir51b^1^* | | 1.06×10^-4^ | | 1.48×10^-6^ | 0.216 | 0.482 |
| *Ir76b^1^* | | 2.08×10^-7^ | | 0.002 | 0.781 | 0.666 |
| *Ir94a^1^* | | 6.82×10^-5^ | | 6.30×10^-6^ | 0.229 | 0.722 |
| *Ir94h^1^* | | 4.24×10^-10^ | | 1.22×10^-6^ | 0.266 | 0.704 |

| **Genotype** | | **PI** | | | | |
| --- | --- | --- | --- | --- | --- | --- |
|  |  | **2.69**  **GA** | **3.50**  **GA** | **5.00**  **GA** | **7.00**  **GA** |  |
| control | 0.56 ± 0.06 | | 0.44 ± 0.07 | 0.22 ± 0.03 | -0.35 ± 0.08 |  |
| *Ir25a^2^* | -0.04 ± 0.04 | | 0.07 ± 0.07 | 0.02 ± 0.07 | -0.24 ± 0.04 |  |
| *Ir51b^1^* | 0.12 ± 0.06 | | -0.12 ± 0.05 | -0.07 ± 0.03 | -0.21 ± 0.04 |  |
| *Ir76b^1^* | -0.23 ± 0.06 | | -0.07 ± 0.04 | -0.07 ± 0.06 | -0.15 ± 0.03 |  |
| *Ir94a^1^* | -0.30 ± 0.06 | | 0.01 ± 0.03 | -0.03 ± 0.09 | -0.12 ± 0.09 |  |
| *Ir94h^1^* | -0.37 ± 0.05 | | 0.01 ± 0.03 | -0.02 ± 0.06 | -0.13 ± 0.08 |  |
|  | | ***P* values** | | | | |
| control | | - | | - | - | - |
| *Ir25a^2^* | | 3.03×10^-6^ | | 0.002 | 0.393 | 0.914 |
| *Ir51b^1^* | | 5.35×10^-4^ | | 2.90×10^-6^ | 0.074 | 0.798 |
| *Ir76b^1^* | | 1.14×10^-8^ | | 1.40×10^-5^ | 0.071 | 0.474 |
| *Ir94a^1^* | | 1.64×10^-9^ | | 2.32×10^-4^ | 0.178 | 0.320 |
| *Ir94h^1^* | | 2.54×10^-10^ | | 2.31×10^-4^ | 0.210 | 0.349 |
